# Virginia State Dental Association

**Published:** 1885-11

**Authors:** 


ARTICLE VII.

### VIRGINIA STATE DENTAL ASSOCIATION.

[Synopsis of Proceedings reported by Dr. E. P. Beadles, of Danville, Va. for THE AMERICAN JOURNAL OF DENTAL SCIENCE.]

The Virginia State Dental Association held its Sixteenth Annual Meeting in the city of Richmond, October 6th, 1885. Dr. Woodley, of Norfork, presiding. The subject of the President's Annual Address was "Professional Excellence in Dentistry." The subject was well handled, and all present were well pleased as well as profited.

Dr. Thackston, of Farmville, read an excellent paper on

"Dental Education" which was highly commended. Dr. Gingrich, of Norfolk, read a paper on "Operative Dentistry." Other papers of interest were read. Clinics were held each day, and several cases of congenital cleft palate were exhibited. Cocaine and many other subjects were up for discussion. It was moved that the "Southern Dental Association" be invited to Virginia, also that the following delegates be appointed to attend its next meeting which takes place in Nashville, Tenn. Drs. Moore, Woodley, Beadles, Mahoney, Bacon, and Faucett.

The following officers were elected, by ballot, for the ensuing term.

W. H. Gingrich, of Norfolk, *President:* E. P. Parsons, of Lynchburg, *First Vice-President:* A. C. Smith, of Louisa, *Second Vice-President:* E. P. Beadles, of Danville, *Third Vice-President:* J. F. Thomson, of Fredericksburg, *Treasurer:* Geo. F. Keesee, of Richmond, *Recording Secretary:* J. Hall Moore, of Richmond, *Corresponding Secretary.*

*Executive Committee:* The following were selected as members of the executive committee. Drs. W. L. Bacon, T. E. Craddock, and F. A. Lee. The meeting was highly interesting and instructive to all. The Association is in a flourishing condition, there being more new members admitted this year than at any previous annual meeting. Those who have stood by the Association when there seemed little to encourage its further existence rejoice that the prospects for its future prosperity are better than they have ever been in the past.

Natural Bridge, of Virginia, has been selected as the place for the next annual meeting, which takes place the second Wednesday in August, 1886.

---

ARTICLE VIII.

